# Diabetic Ketoacidosis and Necrotizing Soft Tissue Infection

**DOI:** 10.21980/J89M0K

**Published:** 2025-04-30

**Authors:** Matthew Henschel, Stephanie Songey

**Affiliations:** * Sutter Roseville Medical Center, Department of Emergency Medicine, Roseville, CA; ^ Sutter Davis Hospital, Department of Emergency Medicine, Davis, CA

## Abstract

**Audience:**

Emergency medicine (EM) residents at all levels of education and medical students on EM rotation.

**Introduction:**

Diabetes is a chronic disease diagnosed in over 28 million people in the United States which causes serious acute complications and is responsible for more than two million ED visits per year.^1^,^2^ Diabetic ketoacidosis (DKA) is one of the most serious complications of diabetes; it is diagnosed with the triad of hyperglycemia, anion gap metabolic acidosis, and ketonemia. The most common cause of DKA is infection, but it can also be precipitated by medication noncompliance, cerebral vascular accident or transient ischemic attack, myocardial infarction, acute pancreatitis, new onset diabetes, and medication side effect, among other causes. Our case involves a patient in DKA that was precipitated by a severe life- and-limb-threatening, necrotizing, soft tissue infection (NSTI). Management includes prompt recognition, antimicrobial therapy, and surgical debridement.^3^

**Educational Objectives:**

At the end of this oral board session, examinees will: 1) Demonstrate the ability to obtain a complete medical history and physical exam. 2) Identify and appropriately treat DKA. 3) Identify, treat, and make appropriate consults for NSTI. 4) Demonstrate effective communication of the treatment plan with the patient.

**Educational Methods:**

This is an oral board case following a standard American Board of Emergency Medicine-style case in a tertiary care hospital with access to all specialists and resources needed.

**Research Methods:**

This case was tested using 12 resident volunteers ranging from PGY 1 – 2 in an ACGME (Accreditation Council for Graduate Medical Education) accredited emergency medicine program in a virtual video conference setting. Practice candidates were seven PGY1 and five PGY2 level residents. Scoring measures of the ACGME core competencies were performed by program core faculty using a scale from 1 – 8 using the American Board of Emergency Medicine (ABEM) oral boards standard case rating. A debriefing session followed the case to discuss the critical actions and for the residents to rate their experience.

**Results:**

The average score for practice candidates per level was: PGY1: 4.4, PGY2: 5.7. Average critical action missed per level was: PGY1: 3.3, PGY2: 0.2. All candidates recognized the patient was in DKA, with varied confidence and comfortability in the appropriate potassium and insulin dosing. On average, practice candidates rated the case as 4.81 (1 – 5 Likert scale, 5 being that the case increased their medical knowledge). No significant modifications were made to the case following the practice session.

**Discussion:**

The aim of this case was to identify and treat two life-threatening diagnoses experienced by patients with diabetes, DKA and NSTI. There are many causes of DKA and the clinician should search for precipitating factors. The most common cause of DKA is infection, but it can also be precipitated by medication noncompliance (both in our case). Even with modern advances, diabetic soft tissue infections can progress to NSTI with high mortality at just over 20%.^1^. NSTI presentation is typically swelling, erythema, and pain out of proportion.^3^ Exam findings that lead to a higher index of suspicion of severe infection are bullae, necrosis, crepitus upon palpitations, and sometimes cutaneous anesthesia.^4^ Imaging modalities can help with diagnosis, but lack of air seen within soft tissue should not rule out NSTI. Suspected NSTI are typically polymicrobial and myonecrosis and should be treated with: 1) vancomycin (or linezolid), 2) either piperacillin/tazobactam, ampicillin/sulbactam, or a carbapenem, 3) clindamycin to decrease toxin production.^2^,^4^

Initial treatment of DKA is isotonic fluids, and insulin therapy should be withheld until serum potassium levels are obtained since prolonged serum acidosis can drive potassium intracellularly. Patients with serum potassium ≤3.3mEq/L should receive potassium replacement prior to initiation of insulin. In adults, insulin can be started as a bolus of 0.1 units/kg body weight followed by 0.1 unit/kg per hour infusion. However, some studies have shown no benefit to insulin bolus in adults.^5^–^6^

**Topics:**

Diabetes, diabetic ketoacidosis, necrotizing soft tissue infection, gas gangrene, myonecrosis.

## USER GUIDE

**Table:** 

List of Resources:	
Abstract	31
User Guide	32
For Examiner Only	34
Oral Boards Assessment	41
Stimulus	44
Debriefing and Evaluation Pearls	56

**Learner Audience:**

Medical Students, Interns, Junior Residents, Senior Residents

**Time Required for Implementation:**

Case: 15 minutes

Debriefing: 10 minutes

Learners per instructor: 1–3

**Topics:**

Diabetes, diabetic ketoacidosis, necrotizing soft tissue infection, gas gangrene, myonecrosis.

**Objectives:**

At the end of this oral boards session, learners will:

1. Demonstrate the ability to obtain a complete medical history and physical exam
2. Identify and appropriately treat DKA and NSTI.
3. Identify, treat, and make appropriate consults for DKA and NSTI.
4. Demonstrate effective communication of the treatment plan with the patient.

### Linked objectives, methods and results

The use of oral board case format was used to give residents experience with the oral board examination and advanced didactic learning. When encountering a patient with hyperglycemia, it is important consider DKA and look for a precipitating factor. (Objective 2). After the initial evaluation, the learners must order appropriate laboratory testing, diagnostic imaging, and initial treatment from their focused history and physical examination (Objective 2). After their laboratory tests and imaging results return, the learner should interpret the results by ensuring appropriate antibiotic coverage, obtain a surgical consult, and ensure proper disposition to the intensive care unit (Objective 3). The instructor will provide stimulus during the case to aid in progression of the case and will gage the learner’s level of understanding and gaps in knowledge through assessment.

### Recommended pre-reading for instructor

- Moleny Jr. et al. Diabetic Ketoacidosis. In: Walls R, Hockberger R, Gausche-Hill M, et al, eds. *Rosen’s Emergency Medicine: Concepts and Clinical Practice*. St. Louis: Mosby, 2022. 9^th^ ed. Philadelphia, PA: Elsevier; 2018; 1538–1547.
- Buboltz JB, Murphy-Lavoie HM. Gas Gangrene. [Updated 2023 Jan 30]. In: StatPearls [Internet]. Treasure Island (FL): StatPearls Publishing; 2025 Jan-. Available from: https://www.ncbi.nlm.nih.gov/books/NBK537030/

### Results and tips for successful implementation

Practice candidates were seven PGY1 level and five PGY2 level residents. This was the first formal practice oral boards for this residency program. All candidates were shown an instructional video of the oral board format from American Board of Emergency Medicine (ABEM) prior to rotating through the oral board cases. The case was presented virtually to 12 residents at levels PGY1-2. Immediate feedback was solicited from the learners participating in the case both by verbal discussion and by completing a rating for the case following the debriefing. After the case, the instructor reviewed important topics with the provided Power-point with a mini lecture, or a focused debrief.

The senior residents did well with this case, and junior residents needed prompting for DKA treatment. It was well received by the learners with a score of 4.81 on a Likert scale (standard scale 1–5 with 5 being excellent) stating it increased their knowledge.

Scoring measures of the ACGME core competencies were performed using ABEM oral exam scoring on a scale from 1 – 8, 1–4 being unacceptable performance and 5 – 8 being acceptable. Efficacy was assumed based on full completion of the case by the residents who acted as practice oral board candidates, and a debriefing session followed to discuss the key components of the case.

The average score for practice candidates per level was: PGY1: 4.4, PGY2: 5.7. Average critical action missed per level was: PGY1: 3.3, PGY2: 0.2. A thorough history would have demonstrated non-adherence to insulin regimen, which should prompt testing of a point of care or serum glucose. A high point of care glucose, or serum glucose, should prompt further investigation with blood gas and metabolic panel for identification of DKA. The most frequent critical action missed was appropriate antibiotic selection. Six of the twelve residents missed no critical actions. Many residents did not consider NSTI immediately because a thorough physical exam of the patient’s foot was not performed, though many residents did voice a concern for NSTI after viewing the foot x-ray. Only one learner missed the identification of gas on the foot x-ray. All candidates recognized the patient was in DKA, with varied confidence and comfortability regarding the appropriate potassium and insulin dosing. Many residents needed prompting on communication with and reevaluating the patient. We feel this was due to inexperience of oral board-style case communication. Feedback from learners was generally positive, with only two learners rating the case as not increasing their medical knowledge.

This case was designed for learners to prepare for EM oral boards as part of the didactic teaching curriculum. After the case was finished, a debriefing session was given to address the critical actions, proper treatment, and disposition.

## Supplementary Information



## Figures and Tables

**Table:** 

**Vitals:**	HR 116	BP 128/76	RR 22/min	Temp 39.1 CO2Sat 97% on room air

**Table:** 

	Milestone	Did not achieve level 1	Level 1	Level 2	Level 3
**1**	**Emergency Stabilization (PC1)**	□ **Did not achieve Level 1**	□ **Recognizes abnormal vital signs**	□ **Recognizes an unstable patient, requiring intervention** **Performs primary assessment** **Discerns data to formulate a diagnostic impression/plan**	□ **Manages and prioritizes critical actions in a critically ill patient** **Reassesses after implementing a stabilizing intervention**
**2**	**Performance of focused history and physical (PC2)**	□ **Did not achieve Level 1**	□ **Performs a reliable, comprehensive history and physical exam**	□ **Performs and communicates a focused history and physical exam based on chief complaint and urgent issues**	□ **Prioritizes essential components of history and physical exam given dynamic circumstances**
**3**	**Diagnostic studies (PC3)**	□ **Did not achieve Level 1**	□ **Determines the necessity of diagnostic studies**	□ **Orders appropriate diagnostic studies** **Performs appropriate bedside diagnostic studies/procedures**	□ **Prioritizes essential testing** **Interprets results of diagnostic studies** **Considers risks, benefits, contraindications, and alternatives to a diagnostic study or procedure**
**4**	**Diagnosis (PC4)**	□ **Did not achieve Level 1**	□ **Considers a list of potential diagnoses**	□ **Considers an appropriate list of potential diagnosis** **May or may not make correct diagnosis**	□ **Makes the appropriate diagnosis** **Considers other potential diagnoses, avoiding premature closure**
**5**	**Pharmacotherapy (PC5)**	□ **Did not achieve Level 1**	□ **Asks patient for drug allergies**	□ **Selects an appropriate medication for therapeutic intervention, considering potential adverse effects**	□ **Selects the most appropriate medication(s) and understands mechanism of action, effect, and potential side effects** **Considers and recognizes drug-drug interactions**
**6**	**Observation and reassessment (PC6)**	□ **Did not achieve Level 1**	□ **Reevaluates patient at least one time during the case**	□ **Reevaluates patient after most therapeutic interventions**	□ **Consistently evaluates the effectiveness of therapies at appropriate intervals**
**7**	**Disposition (PC7)**	□ **Did not achieve Level 1**	□ **Appropriately selects whether to admit or discharge the patient**	□ **Appropriately selects whether to admit or discharge** **Involves the expertise of some of the appropriate specialists**	□ **Educates the patient appropriately about their disposition** **Assigns patient to an appropriate level of care (ICU/Tele/Floor)** **Involves expertise of all appropriate specialists**
**22**	**Patient centered communication (ICS1)**	□ **Did not achieve level 1**	□ **Establishes rapport and demonstrates empathy to patient (and family)** **Listens effectively**	□ **Elicits patient’s reason for seeking health care**	□ **Manages patient expectations in a manner that minimizes potential for stress, conflict, and misunderstanding**.
**23**	**Team management (ICS2)**	□ **Did not achieve level 1**	□ **Recognizes other members of the patient care team during case (nurse, techs)**	□ **Communicates pertinent information to other healthcare colleagues**	□ **Communicates a clear, succinct, and appropriate handoff with specialists and other colleagues** **Communicates effectively with ancillary staff**

**Table:** 

**#1**	**Patient Information Form**
**#2**	**Venous Blood gas**
**#3**	**Complete Blood Count**
**#4**	**Basic Metabolic Panel and beta-**hydroxybutyrate **level**
**#5**	**Urinalysis**
**#6**	**Monitor Strip**
**#7**	**12 Lead EKG**
**#8**	**Chest X-ray**
**#9**	**Foot X-ray**
**#10**	**Point of Care Blood Glucose**

**Table:** 

**Patient’s Name:**	**George Takis**
**Age:**	**49 years old**
**Gender:**	**Male**
**Chief Complaint:**	**Right foot pain**
**Person Providing History:**	**Patient**
**Vital Signs:**	
** Temp:**	**39.1°C (102.4°F)**
** BP:**	**128/76**
** P:**	**116 HR**
** RR:**	**22 RR**
** Pulse Ox:**	**97% on room air**
** Weight:**	**90kg (198lbs)**
** Height:**	**65 inches**

**Table:** 

**pH**	**7.22**
**pCO2**	**37 mmHg**
**pO2**	**37 mmHg**
**O2 saturation**	**74%**
**Lactate**	**3.6 mg/dL**

**Table:** 

**WBC**	**16 × 10** **^3^** **/μL**
**Hgb**	**11.4 g/dL**
**Hct**	**34.2%**
**Platelets**	**275 × 10** **^3^** **/μL**
**Differential**	
** Neutrophils**	**68%**
** Lymphocytes**	**17%**
** Monocytes**	**22%**
** Eosinophils**	**2%**
** Bands**	**1%**

**Table:** 

**Sodium**	**130 mEq/L**
**Potassium**	**3.5 mEq/L**
**Chloride**	**94 mEq/L**
**Carbon Dioxide**	**12 mEq/L**
**Blood urea nitrogen**	**36 mg/dL**
**Creatinine**	**2.0 mg/dL**
**Glucose**	**862 mg/dL**
**Serum beta-hydroxybutyrate**	**>3 mEq/L**

**Table:** 

**Appearance**	**Clear**
**Color**	**Yellow**
**Glucose**	**>1000 mg/dL**
**Ketones**	**80 mg/dL**
**Sp Gravity**	**>1.030**
**Blood**	**negative**
**pH**	**6.0**
**Protein**	**negative**
**Nitrite**	**negative**
**Leukocyte**	**negative**
**WBC**	**0–1/hpf**
**RBC**	**0–2/hpf**
**Squamous Cells**	**0/hpf**
**Bacteria**	**0/hpf**

**Figure:**
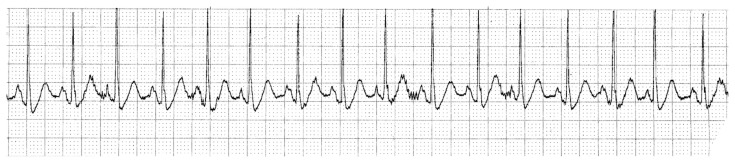
Author’s own images

**Figure:**
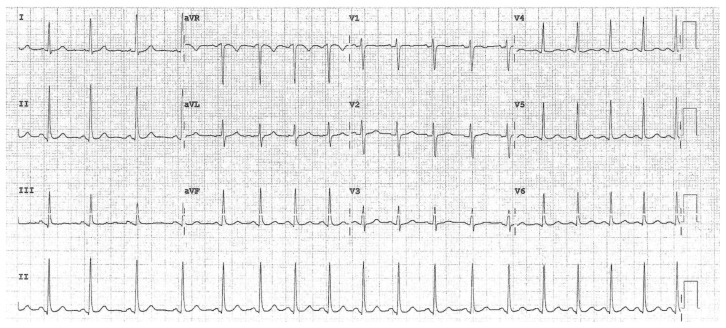
Author’s own images

**Figure:**
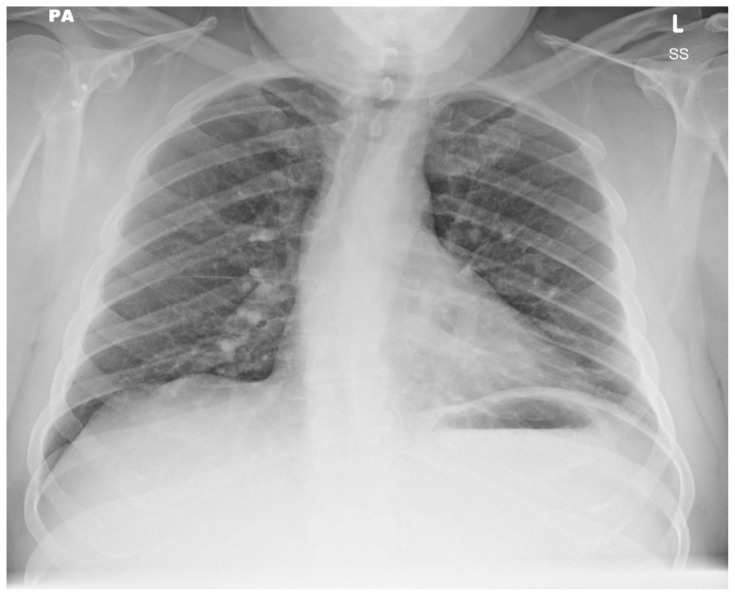
Author’s own images

**Figure:**
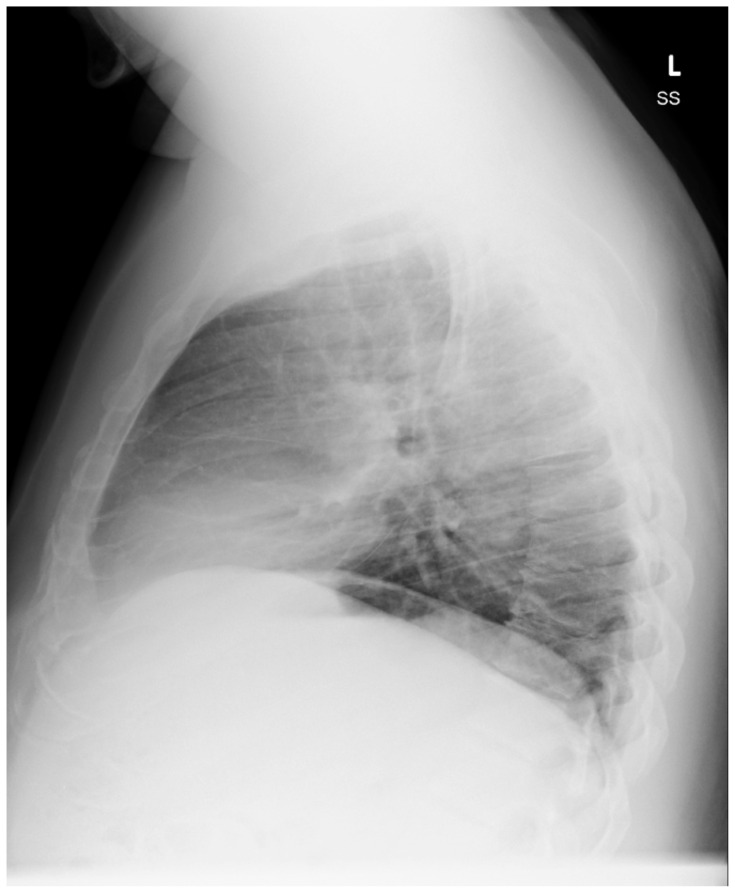
Author’s own images

**Figure:**
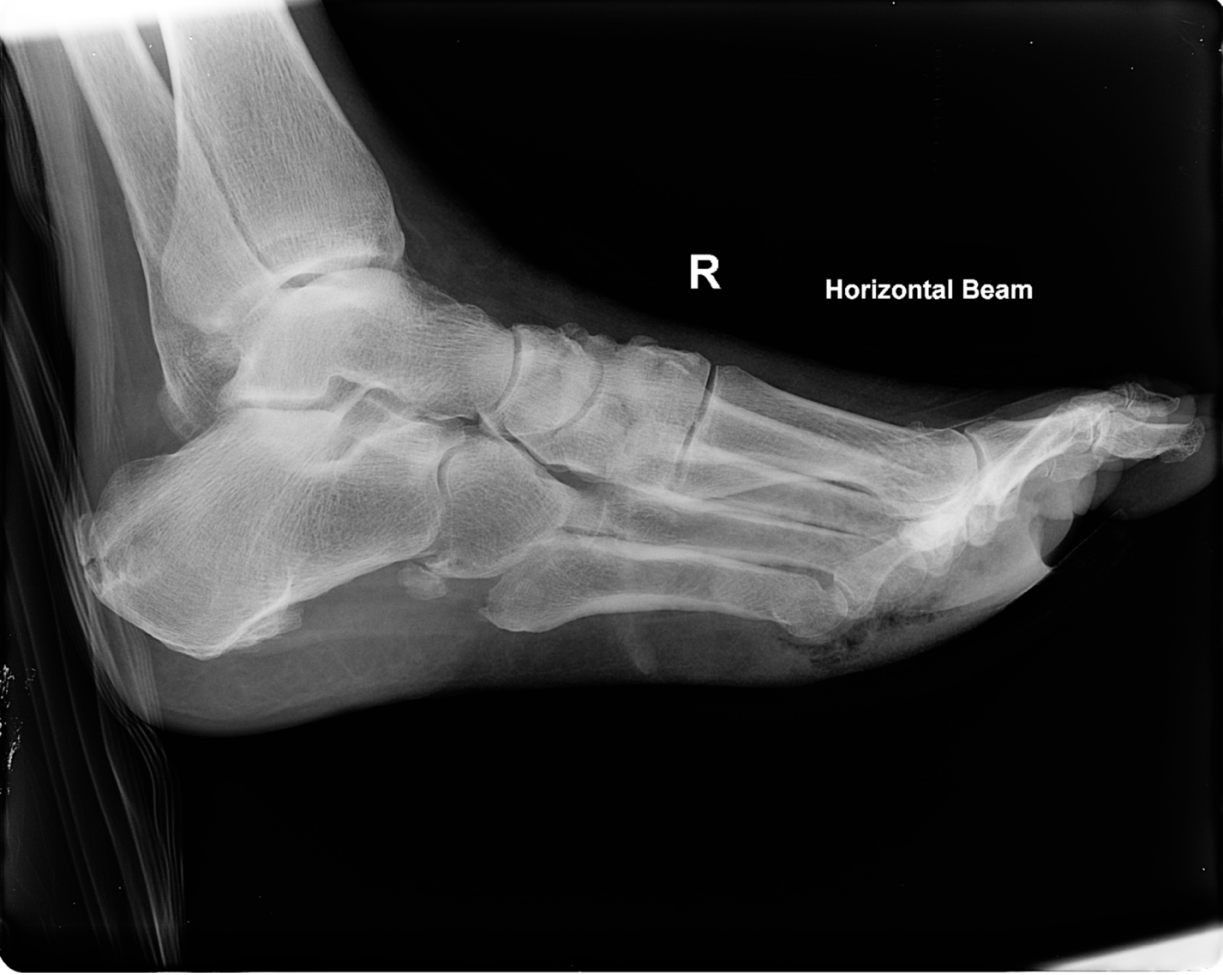


**Figure:**
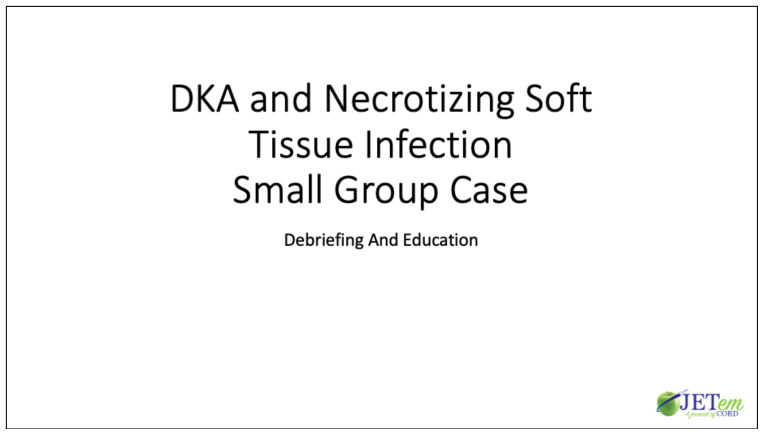
Please see associated PowerPoint file

## FOR EXAMINER ONLY

### Oral Case Summary

**Diagnosis: Diabetic Ketoacidosis and Necrotizing Soft Tissue Infection**

**Case Summary:** A 49-year-old male with a past medical history of Type 1 diabetes presents to the ED with right foot pain that started three days ago. He has had similar pain in the past that has required surgical intervention. Due to recent relocation, the patient has not yet established care with a new physician and ran out of his insulin 10 days ago. He has not checked his temperature and has not been checking his glucose levels. His vital signs show tachycardia, tachypnea, and he is febrile. The physical exam is significant with right foot edema and erythema on the dorsal surface which is beginning to extend above the ankle with associated crepitus. Appropriate evaluation then reveals the patient is in DKA, requiring an insulin drip, as well as NSTI of the right foot requiring surgical consultation, antibiotic therapy, and ICU admission.

**Order of Case:** Patient arrives in a private vehicle by himself. The patient is triaged; vital signs are performed. The learner will assess for appropriate airway, breathing, and circulation, finding the patient to be tachycardic. At this time, the learner should ask nursing team to place the patient on a cardiac monitor, start an IV, check point-of-care glucose, and start fluids. It is acceptable if the learner addresses the vital signs with fluids, or waits until a history is taken. The physical exam is performed and includes a thorough right foot evaluation, with (extra points if learner identifies crepitus). The learner should request the nurse to start an IV line if not done so already, appropriately identifying SIRS criteria and concern for sepsis. Any isotonic fluids are appropriate at the standard dose for sepsis, 30 ml/kg. If not already done, the learner also orders a point-of-care glucose, and the nurse reports the result just reads “high.” The learner then orders appropriate labs which should include CBC, BMP, serum osmolality, serum beta-hydroxybutyrate, venous blood gas (VBG) with lactate, urinalysis, two blood cultures, chest x-ray, right foot X-ray, and electrocardiogram (ECG).

The learner should start the patient on broad spectrum antibiotics. Appropriate treatment is vancomycin (or linezolid) plus piperacillin/tazobactam, meropenem, or imipenem-cilastin. The leaner may wait to add NSTI specific treatment to include clindamycin until after x-ray right foot is resulted. The patient will then go to x-ray; while in x-ray, the patient’s labs return, and the learner should appropriately identify that the patient is in DKA. After interventions, the leaner should reassess vital signs. The learner should order an additional 2L of isotonic crystalloids to run over two hours, with 20 mEq potassium per liter for first 2 liters. Then based on labs maintain 20–40 mEq/hour. The learner then orders insulin drip to start at 9 units per hour (0.1 unit/kg/hr). The learner orders point-of-care glucose every hour and beta-hydroxybutyrate and BMP every 2 hours. The learner reads the ECG. The x-rays are now available and the learner interprets x-rays, hopefully recognizing the gas in the tissues. The learner should recognize that this is probably a clostridial infection and orders the appropriate antibiotics, clindamycin. The learner is allowed to consult a pharmacist for antimicrobials. The learner should update the patient and explain the results of the work up, check tetanus status, order Tdap for patient, and consult a surgeon for emergent surgery. The learner will need to sign the patient out to the intensivist, indicating the patient is critically ill and requires ICU admission. The patient departs for surgery, ending the case.

**Disposition:** Admission to ICU. Immediate surgery with appropriate consultant

**Critical Actions:**

1. Orders BMP & VBG (data acquisition, systems based practice)
2. Hydrates patient in response to DKA (problem solving, patient care)
3. Start Insulin drip after appropriate assessment of electrolytes (problem solving, patient care)
4. Orders & interprets X-ray (data acquisition, problem solving, systems-based practice)
5. Administers appropriate antibiotics for NSTI (patient care)
6. Consults surgical service for immediate surgical management of NSTI. (problem solving, Patient care, clinical competence)
7. Consults intensivist for admission (problem solving, patient care, clinical competence)

### Historical Information

**Chief Complaint:** Right foot pain

**History of Present Illness:** 49-year-old man with known Type 1 Diabetes presents with a chief complaint of right foot pain which began 3 days ago. He has a history of prior surgery on the right foot. He did not take his temperature at home and has not been checking his glucose. He just relocated to the area and does not have a primary physician. He has been out of insulin for ten days.

**Past Medical History:** Diabetes, Type 1

**Past Surgical History:** appendectomy, cholecystectomy, and two previous surgeries on the right foot

**Patient’s Medications:** Novolog Mix 70/30, 32 units with breakfast & 15 units with dinner

**Allergies:** NKDA

**Social history:**

- Smoking: Does not smoke
- Tobacco: Does not use tobacco
- Alcohol: Drinks alcohol occasionally
- Drug use: Does not use illicit drugs

**Family history:**

- Mother – HTN
- Father – hyperlipidemia, CAD
- Sister – no chronic medical conditions

### Physical Exam Information

**Weight:** 90kg

**General appearance:** appears stated age, cooperative, answers questions appropriately, does not appear to be in any acute distress

**Primary survey:**

- **Airway: Patent**
- **Breathing: Tachypneic in no respiratory distress with equal breath sounds**
- **Circulation: tachycardic perfusing appropriately**

**Physical examination: General appearance:** appears his stated age, cooperative, answers questions appropriately, does not appear to be in any acute distress

- **HEENT:**
  ○ **Head:** within normal limits
  ○ **Eyes:** within normal limits
  ○ **Ears:** within normal limits
  ○ **Nose:** within normal limits
  ○ **Oropharynx/Throat:** pharynx clear but mucous membranes are dry, has fruity odor to breath
- **Neck:** within normal limits
- **Chest:** within normal limits
- **Cardiovascular:** apical regular, normal heart tones, slightly tachycardic, pulses palpable and equal
- **Abdominal/GI:** within normal limits
- **Genitourinary:** within normal limits
- **Rectal:** within normal limits
- **Extremities: right foot**: multiple healed scars from previous surgeries, very edematous, warm and erythematous to the superior surface of the foot which is beginning to extend above the ankle. The candidate must ask for findings to palpitation:, there is crepitus, exquisite tenderness to palpation, pulses are palpable and adequate, and sensation is decreased to light touch. He has full range of motion of the ankle and toes. There is no eschar.
- **Back:** within normal limits
- **Neuro:** within normal limits
- **Skin:** within normal limits
- **Lymph:** enlarged nodes in the right groin
- **Psych:** within normal limits

### Critical Actions and Cueing Guidelines

1. **Critical Action #1:** *Orders BMP & VBG*
  (data acquisition, systems based practice)
  This action is met by the learner ordering a BMP or a CMP and VBG or ABG when ordering laboratory testing.
  - Cueing guideline (if applicable):
    *If the learner does not order the BMP and VBG, the nurse says, “I drew some blood, do want me to send it to the lab?”*
2. **Critical Action #2:** *Hydrates patient in response to dehydration*
  (problem solving, patient care)
  This action is met by the learner ordering an IV to be started and orders a bolus of crystalloid. Considering that the patient meets sepsis criteria and the patient has no contraindications, the amount should be 30 ml/kg.
  - Cueing guideline (if applicable):
    *If the learner does not order a bolus of fluid, the patient should state that he is really thirsty and can he have a couple of glasses of water*.
3. **Critical Action #3:** *Start Insulin drip*
  (problem solving, patient care)
  This action is met by the learner ordering an insulin drip at the rate of 0.1 units per kg/hour.
  - Cueing guideline (if applicable):
    *If the learner does not order an insulin drip, the nurse should tell the learner that she has a critical value for him and inform him of the blood glucose level*.
4. **Critical Action #4:** *Orders & interprets x-ray right foot*
  (data acquisition, Problem solving, systems-based practice)
  This action is met by the learner seeing the x-ray of the foot, commenting on the gas and the significance of it.
  - Cueing guideline (if applicable):
    *If the learner does not mention the significance of the gas in the tissues, the patient should ask if he can see the x-ray and then ask questions about what it shows. When the learner explains the x-ray findingso him, the patient should ask “what does that mean?”*
5. **Critical Action #5:** *Administers appropriate antibiotics*
  (patient care)
  This action is met by the learner ordering Vancomycin and another antibiotic (piperacillin-tazobactam, meropenem or imipenem-cilastin, plus clindamycin. The learner is allowed to ask to look this up in a reference, talk to a pharmacist or another consultant to determine this.
  - Cueing guideline (if applicable):
    *If the learner does not order an antibiotic, the nurse should ask if the learner wants to give any medications. If the learner only orders one antibiotic then the nurse should ask, “Do think that is enough for this patient?” Finally, the pharmacist may call and want to discuss the antibiotics. If this prompt is used then the learner should be scored down*.
6. **Critical Action #6:** *Consults ortho or podiatry for immediate surgery*
  (problem solving, Patient care, clinical competence)
  This action is met by the learner requesting to speak to either podiatry or orthopedic surgeon and recommends surgery.
  - Cueing guideline (if applicable):
    *If the learner does not consult the orthopedic surgeon or podiatrist, the intensivist should ask the learner when the report is received, “what do you think we should do about his foot?”*

## ORAL BOARDS ASSESSMENT

*Diabetic Ketoacidosis and Necrotizing Soft Tissue Infection*

Learner: _________________________________________

**Critical Actions:**

□ Orders BMP & VBG
□ Hydrates patient in response to dehydration
□ Start Insulin drip
□ Orders & interprets x-ray right foot
□ Administers appropriate antibiotics
□ Consults ortho or podiatry for immediate surgery

**Summative and formative comments:**

**Milestone assessment:**

### Stimulus Inventory

#### Stimulus #7

##### 12 lead ECG

**PR 126 msec**

**QRS 81 msec**

**QT 314 msec**

**QTc 421 msec**

#### Stimulus #9

##### Foot X-ray

Knipe H. Anaerobic cellulitis. In: Radiopaedia.org https://radiopaedia.org/cases/anaerobic-cellulitis?lang=us CC BY-NC-SA 3.0.

#### Stimulus #10

##### POINT OF CARE BLOOD SUGAR

*****HI*****
