# Supplementary material for: Diabetic Ketoacidosis and Necrotizing Soft Tissue Infection
Source: J Educ Teach Emerg Med. 2025 Apr 30;10(2):O30–56. doi: 10.21980/J89M0K (PMC12054117; doi:10.21980/J89M0K)
Supplement: Supplementary file 1 [file 10-2-O30-supp1.pptx]

## Slide 1
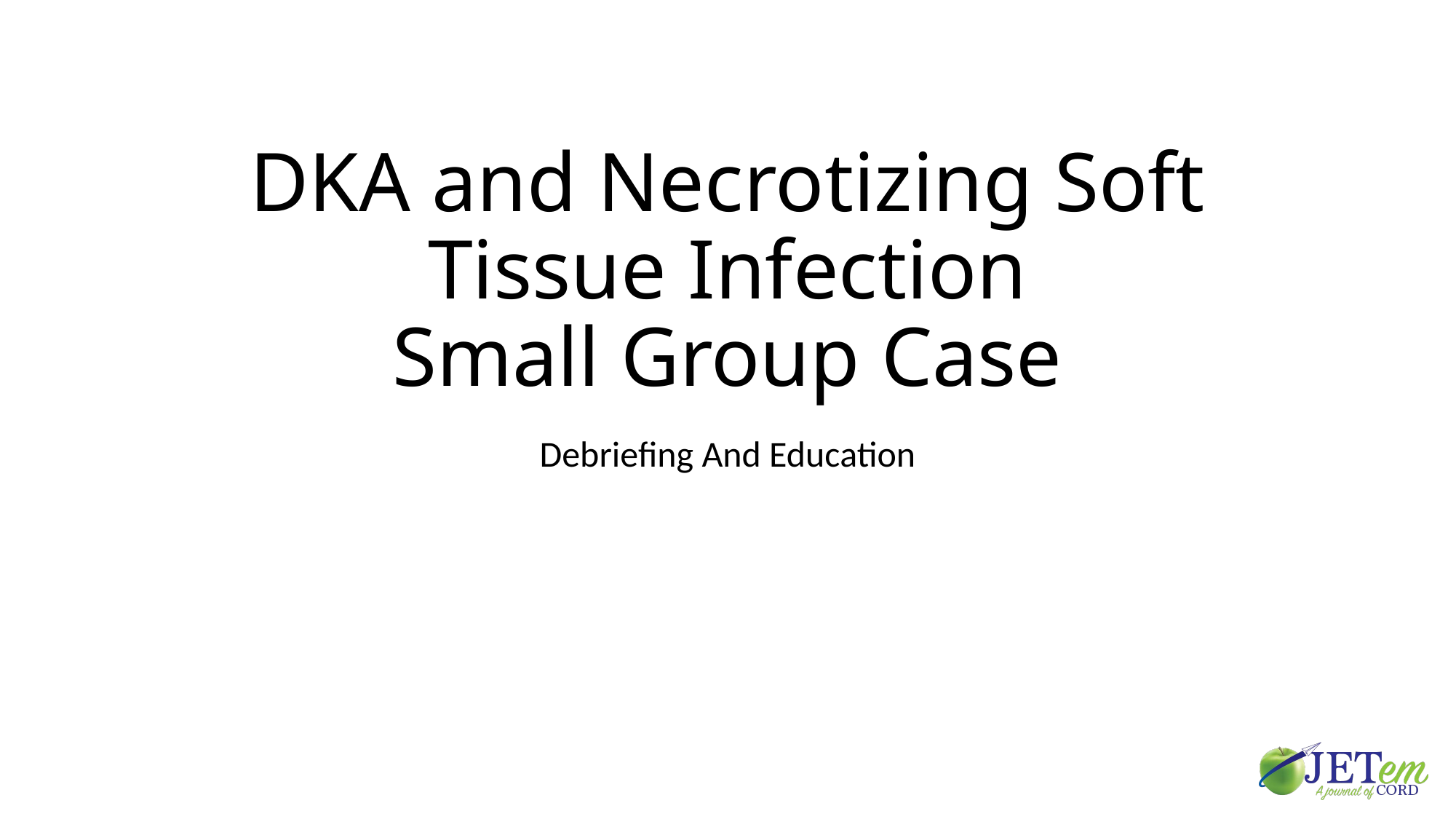

# DKA and Necrotizing Soft Tissue Infection Small Group Case
Debriefing And Education

## Slide 2
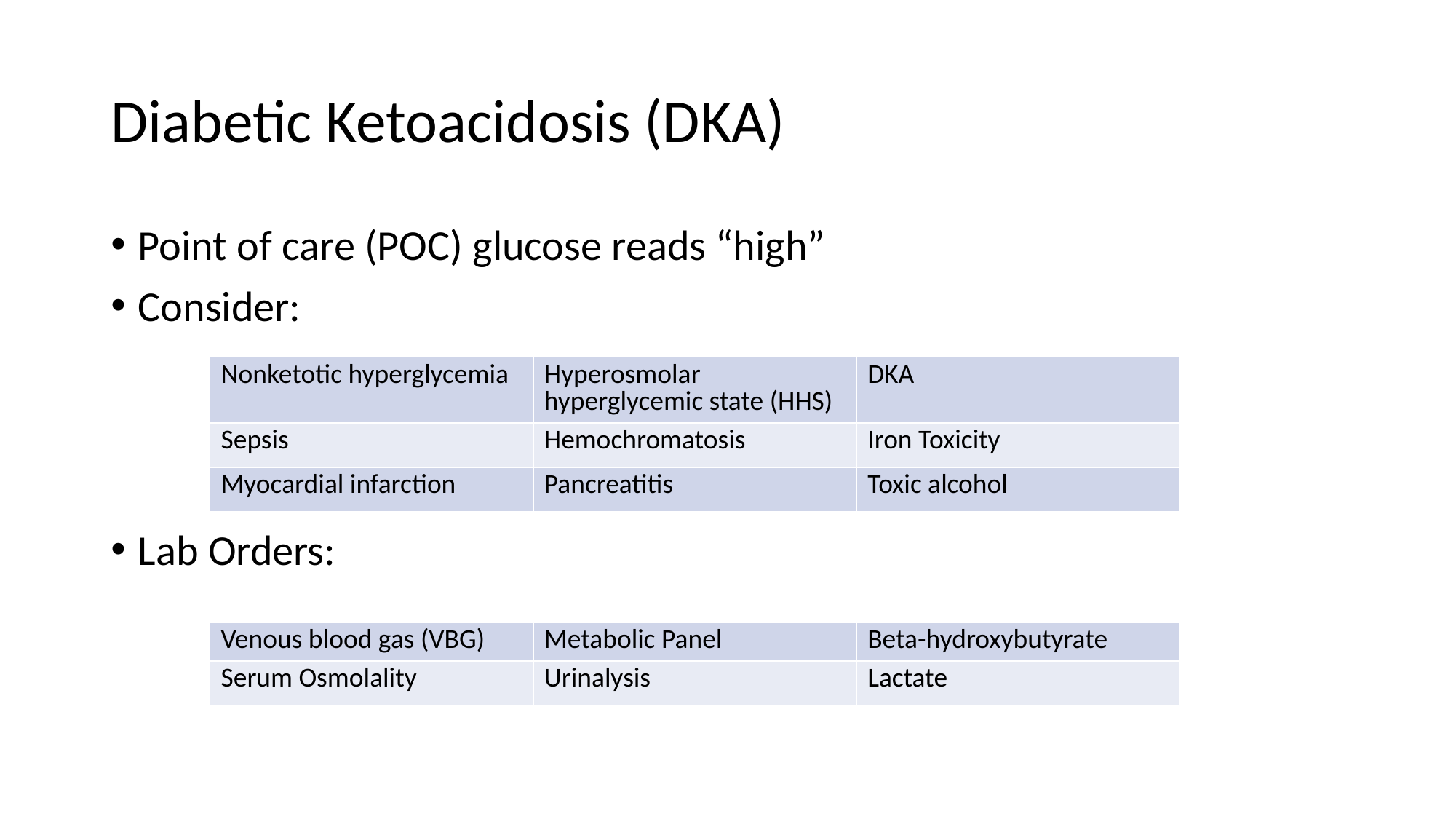

# Diabetic Ketoacidosis (DKA)
Point of care (POC) glucose reads “high”
Consider:
Lab Orders:
| Nonketotic hyperglycemia | Hyperosmolar hyperglycemic state (HHS) | DKA |
| --- | --- | --- |
| Sepsis | Hemochromatosis | Iron Toxicity |
| Myocardial infarction | Pancreatitis | Toxic alcohol |
| Venous blood gas (VBG) | Metabolic Panel | Beta-hydroxybutyrate |
| --- | --- | --- |
| Serum Osmolality | Urinalysis | Lactate |

## Slide 3
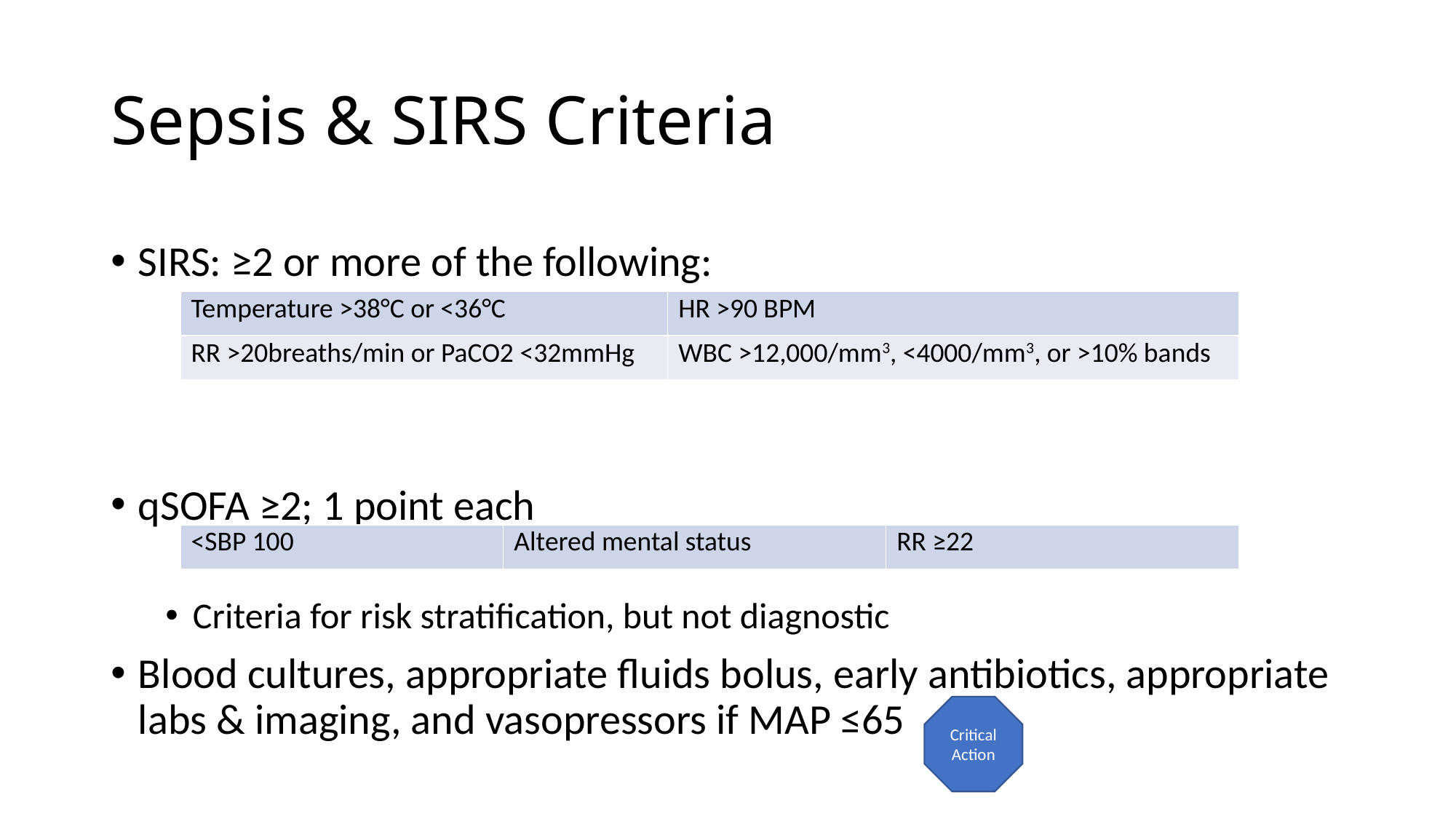

# Sepsis & SIRS Criteria
SIRS: ≥2 or more of the following:
qSOFA ≥2; 1 point each
Criteria for risk stratification, but not diagnostic
Blood cultures, appropriate fluids bolus, early antibiotics, appropriate labs & imaging, and vasopressors if MAP ≤65
| Temperature >38°C or <36°C | HR >90 BPM |
| --- | --- |
| RR >20breaths/min or PaCO2 <32mmHg | WBC >12,000/mm3, <4000/mm3, or >10% bands |
| <SBP 100 | Altered mental status | RR ≥22 |
| --- | --- | --- |
Critical Action

## Slide 4
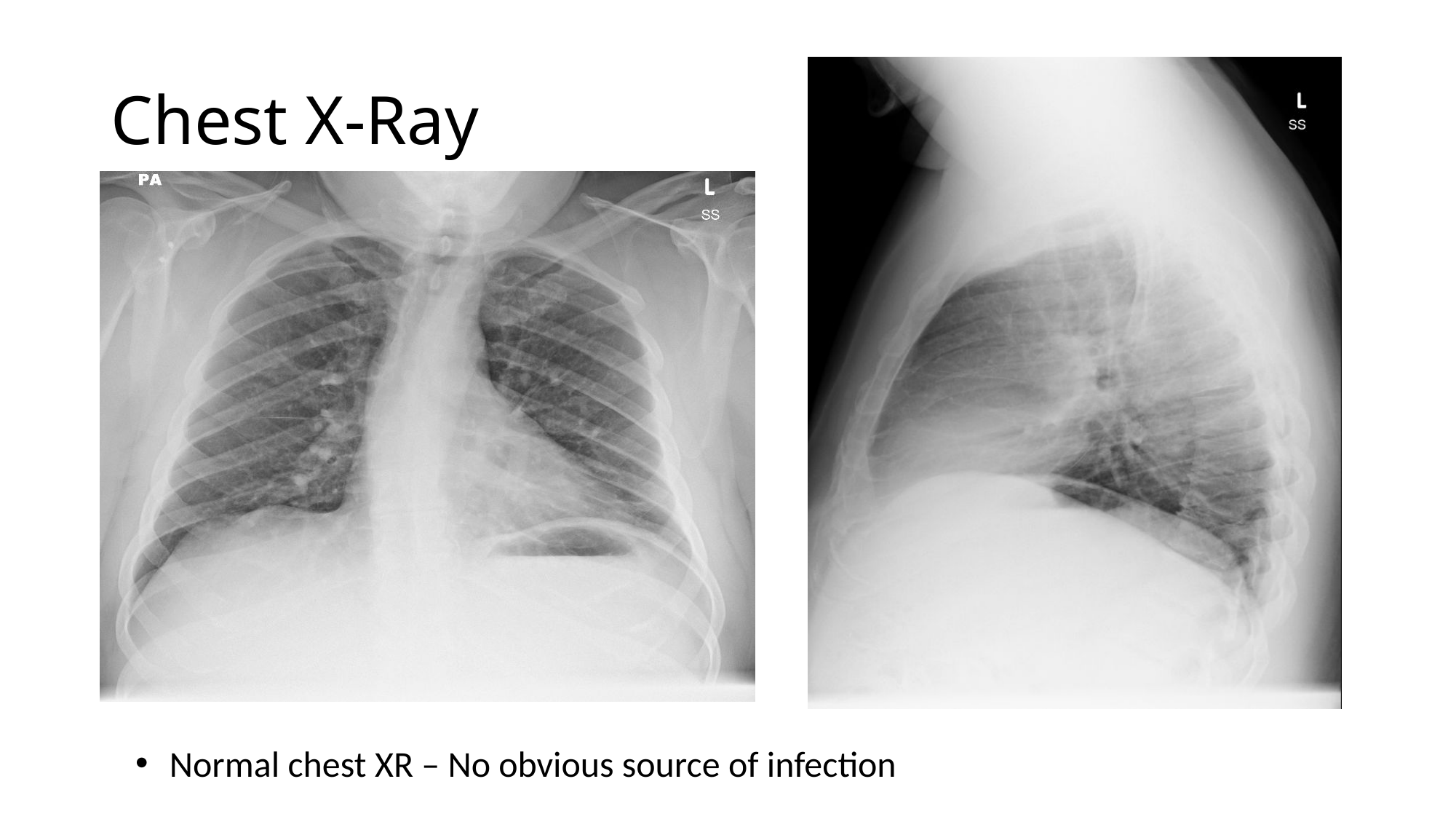

# Chest X-Ray
Normal chest XR – No obvious source of infection

## Slide 5
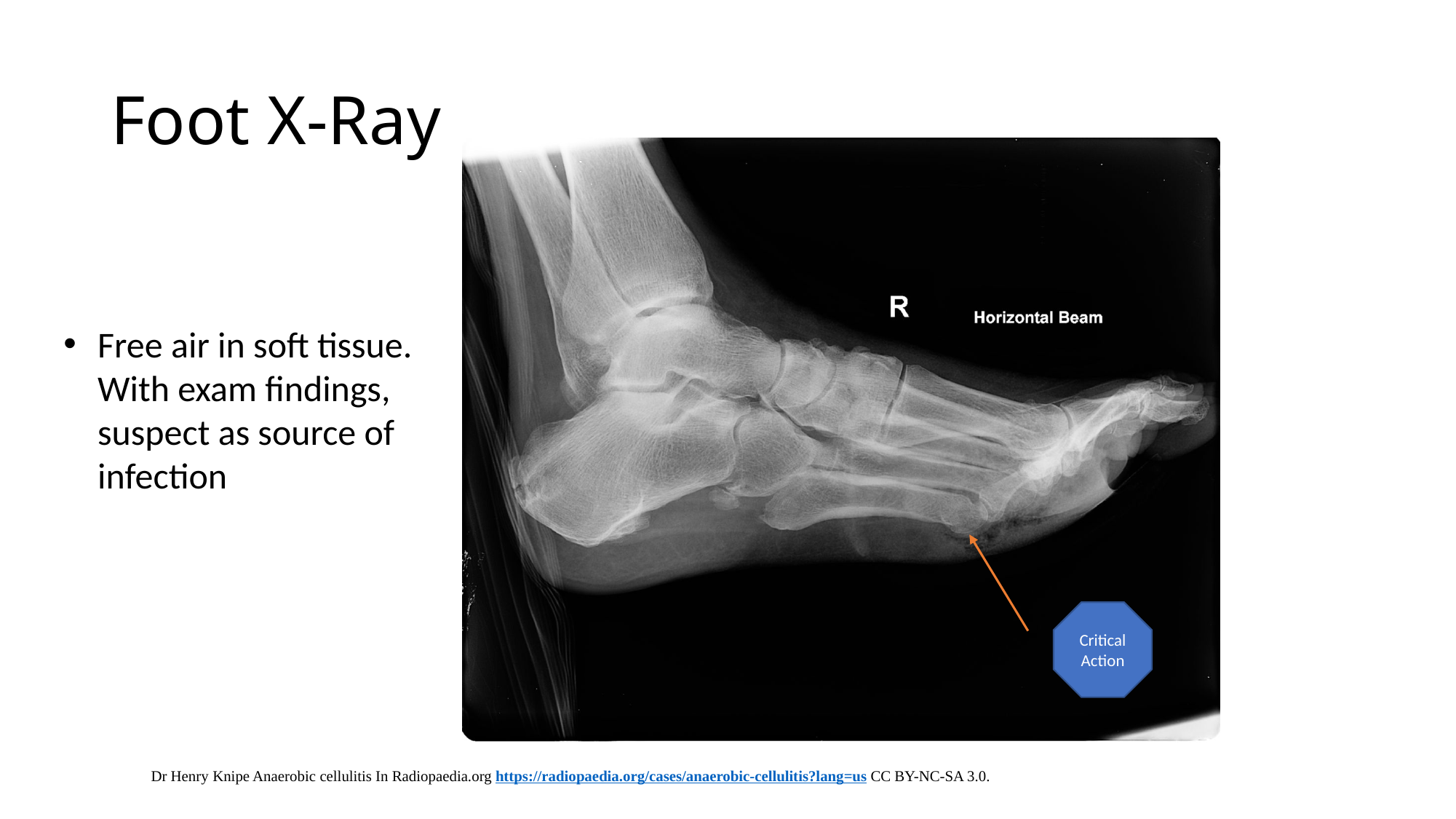

# Foot X-Ray
Free air in soft tissue. With exam findings, suspect as source of infection
Critical Action
Dr Henry Knipe Anaerobic cellulitis In Radiopaedia.org https://radiopaedia.org/cases/anaerobic-cellulitis?lang=us CC BY-NC-SA 3.0.

## Slide 6
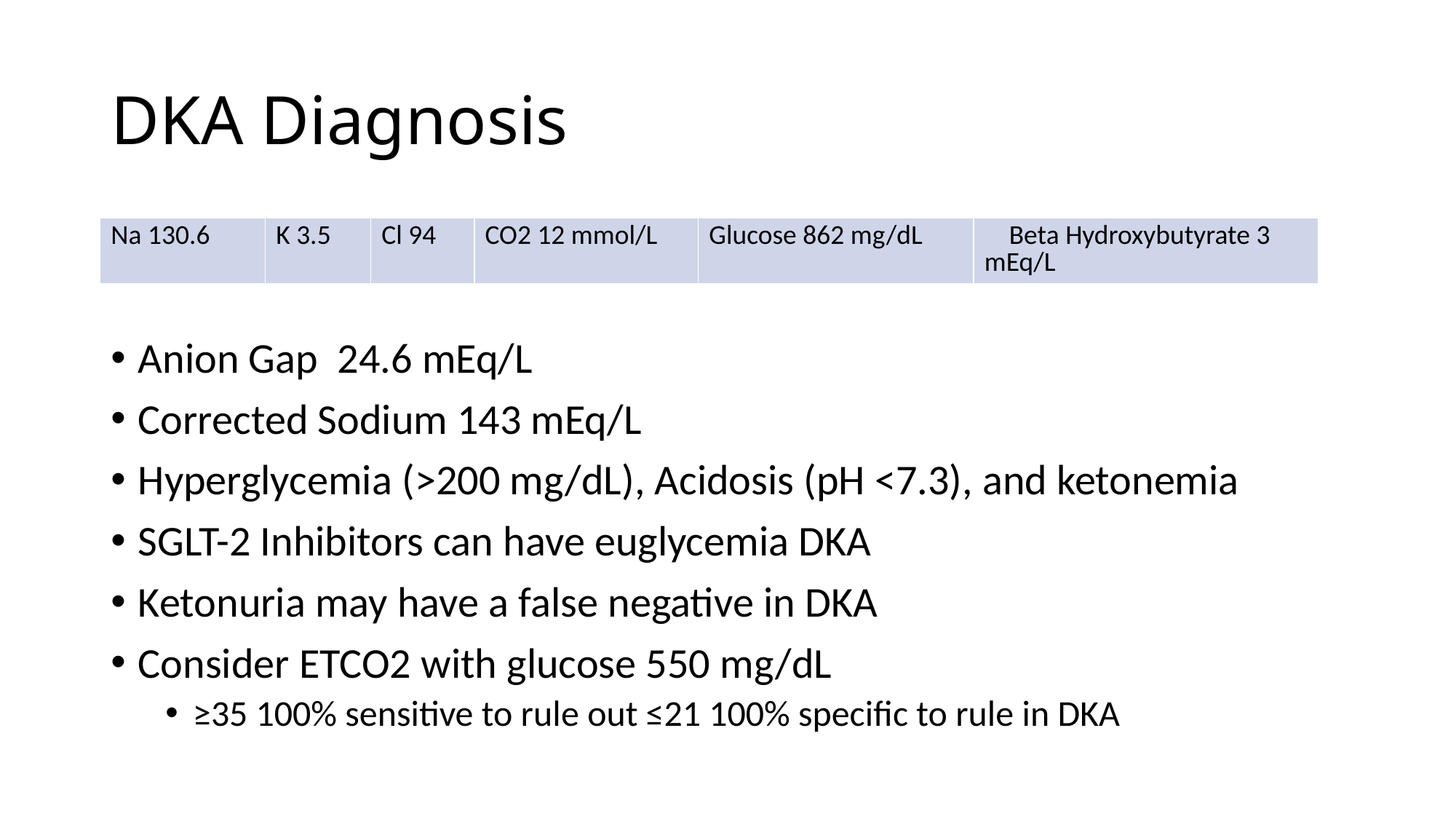

# DKA Diagnosis
| Na 130.6 | K 3.5 | Cl 94 | CO2 12 mmol/L | Glucose 862 mg/dL | Beta Hydroxybutyrate 3 mEq/L |
| --- | --- | --- | --- | --- | --- |
Anion Gap 24.6 mEq/L
Corrected Sodium 143 mEq/L
Hyperglycemia (>200 mg/dL), Acidosis (pH <7.3), and ketonemia
SGLT-2 Inhibitors can have euglycemia DKA
Ketonuria may have a false negative in DKA
Consider ETCO2 with glucose 550 mg/dL
≥35 100% sensitive to rule out ≤21 100% specific to rule in DKA

## Slide 7
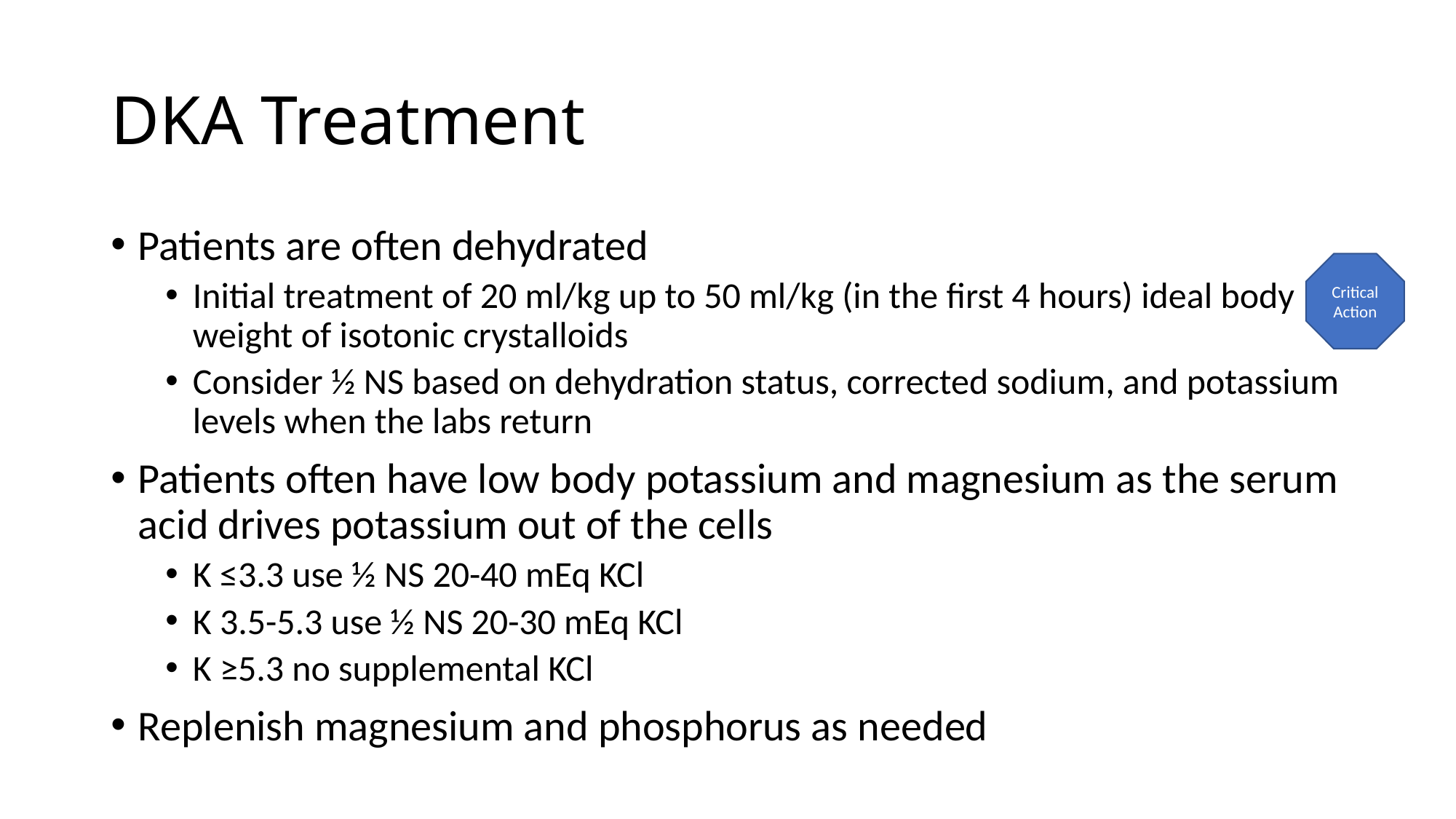

# DKA Treatment
Patients are often dehydrated
Initial treatment of 20 ml/kg up to 50 ml/kg (in the first 4 hours) ideal body weight of isotonic crystalloids
Consider ½ NS based on dehydration status, corrected sodium, and potassium levels when the labs return
Patients often have low body potassium and magnesium as the serum acid drives potassium out of the cells
K ≤3.3 use ½ NS 20-40 mEq KCl
K 3.5-5.3 use ½ NS 20-30 mEq KCl
K ≥5.3 no supplemental KCl
Replenish magnesium and phosphorus as needed
Critical Action

## Slide 8
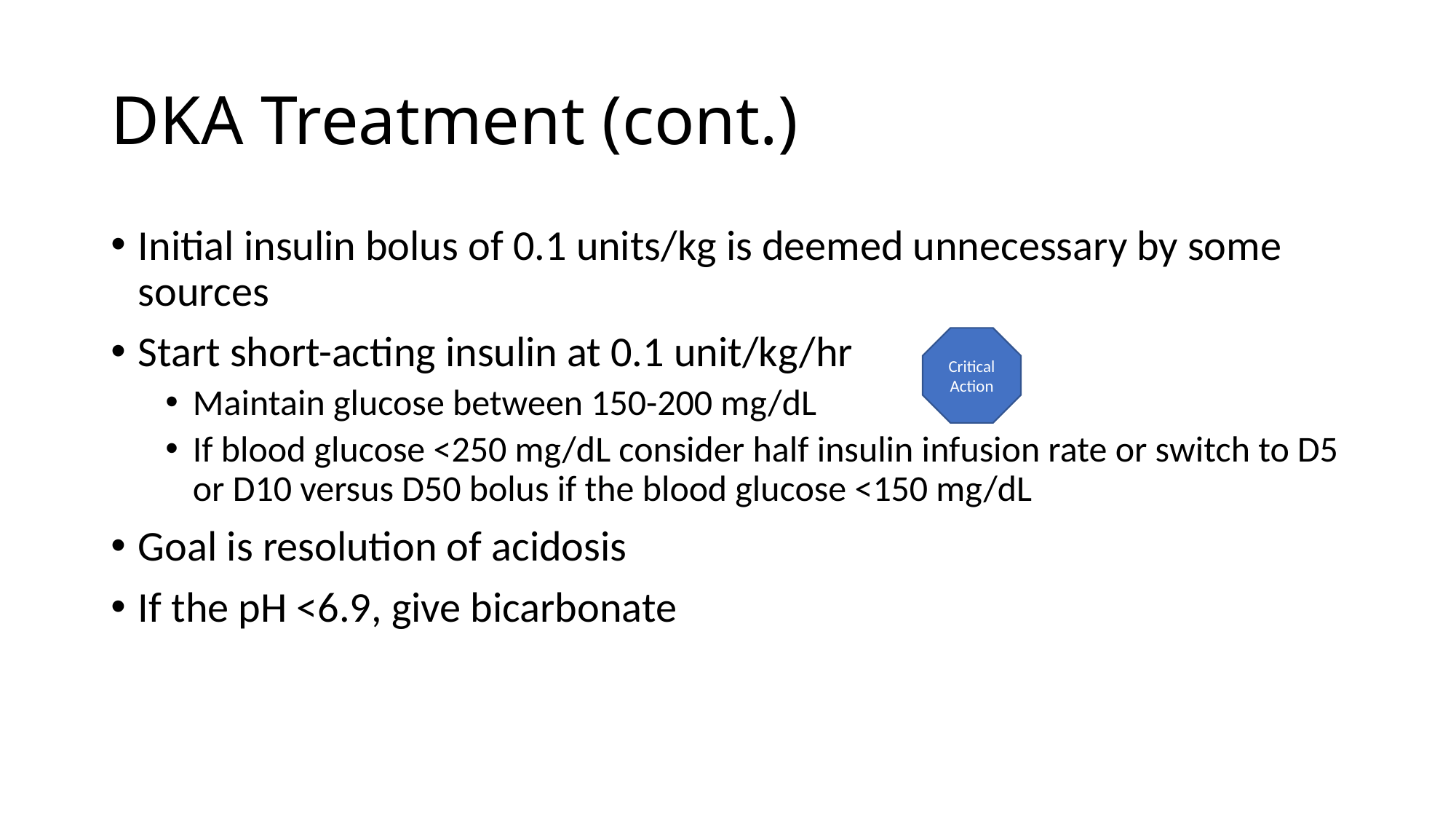

# DKA Treatment (cont.)
Initial insulin bolus of 0.1 units/kg is deemed unnecessary by some sources
Start short-acting insulin at 0.1 unit/kg/hr
Maintain glucose between 150-200 mg/dL
If blood glucose <250 mg/dL consider half insulin infusion rate or switch to D5 or D10 versus D50 bolus if the blood glucose <150 mg/dL
Goal is resolution of acidosis
If the pH <6.9, give bicarbonate
Critical Action

## Slide 9
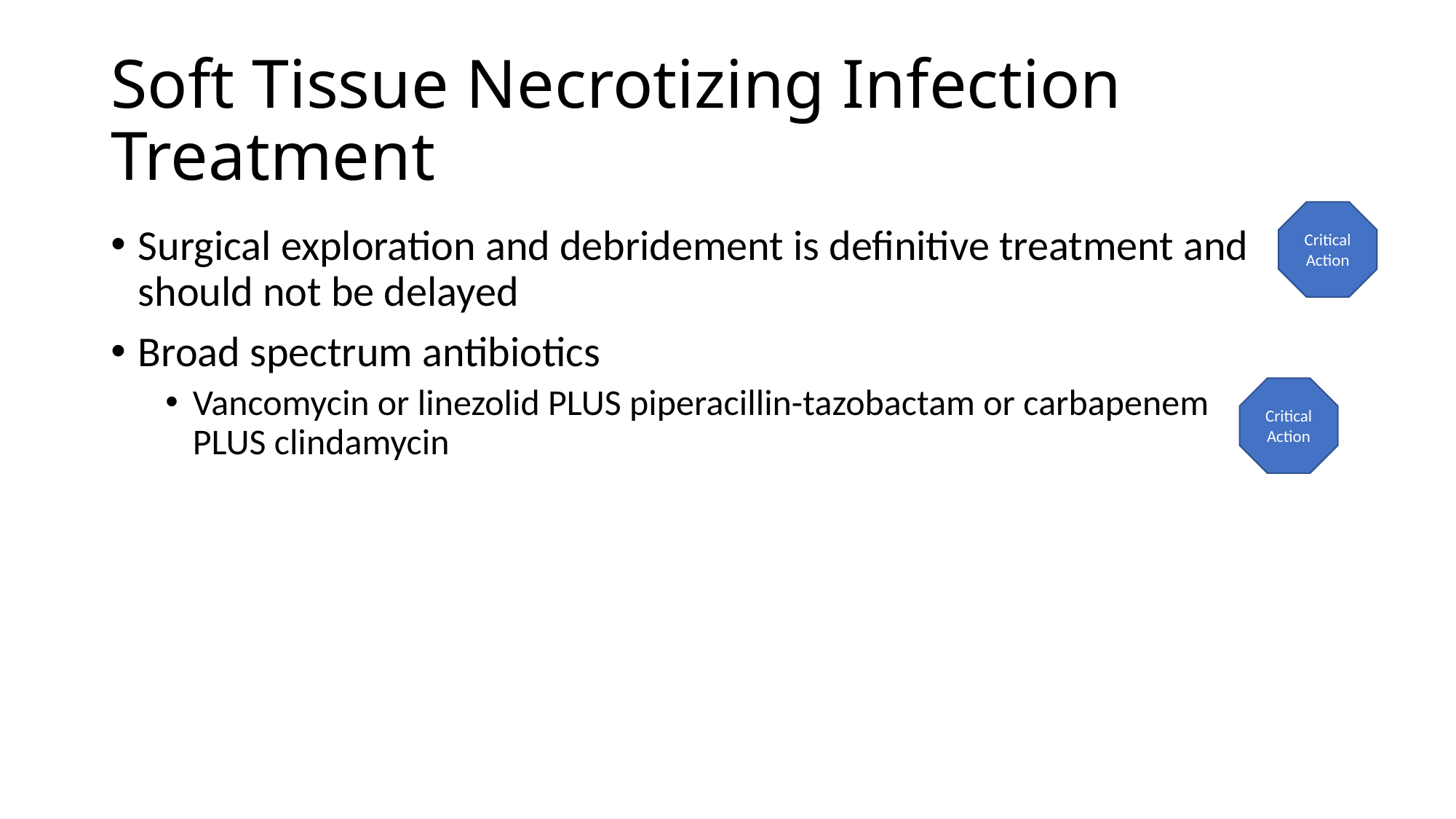

# Soft Tissue Necrotizing Infection Treatment
Critical Action
Surgical exploration and debridement is definitive treatment and should not be delayed
Broad spectrum antibiotics
Vancomycin or linezolid PLUS piperacillin-tazobactam or carbapenem PLUS clindamycin
Critical Action

## Slide 10
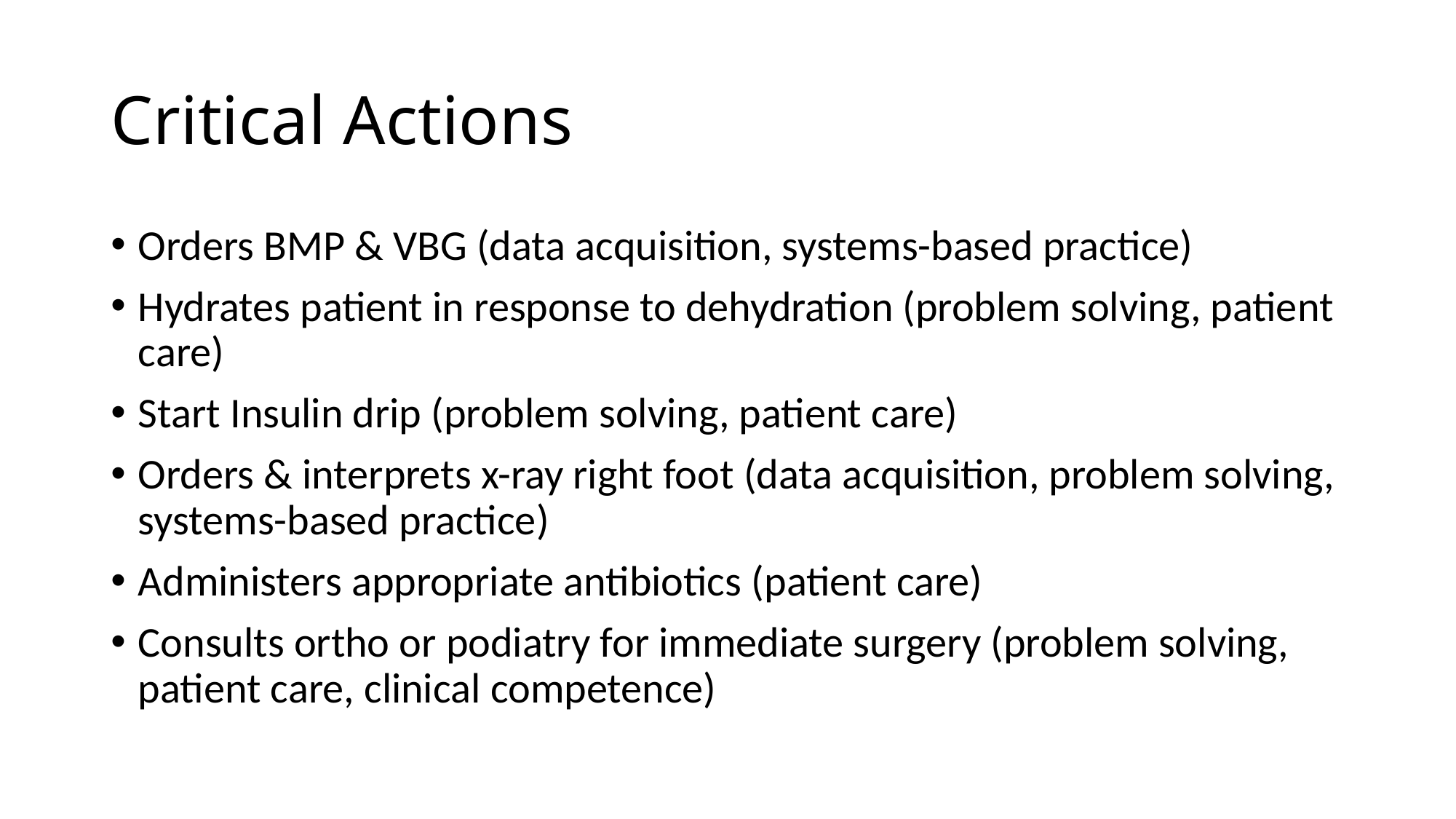

# Critical Actions
Orders BMP & VBG (data acquisition, systems-based practice)
Hydrates patient in response to dehydration (problem solving, patient care)
Start Insulin drip (problem solving, patient care)
Orders & interprets x-ray right foot (data acquisition, problem solving, systems-based practice)
Administers appropriate antibiotics (patient care)
Consults ortho or podiatry for immediate surgery (problem solving, patient care, clinical competence)
